# Supplementary material for: A noninvasive fluorescence imaging-based platform measures 3D anisotropic extracellular diffusion
Source: Nat Commun. 2021 Mar 26;12:1913. doi: 10.1038/s41467-021-22221-0 (PMC7997923; doi:10.1038/s41467-021-22221-0)
Supplement: Supplementary file 1 — Supplementary Information [file 41467_2021_22221_MOESM1_ESM.pdf]

SUPPLEMENTARY INFORMATION

A noninvasive fluorescence imaging-based platform measures 3D anisotropic extracellular diffusion

Peng Chen, Xun Chen, R. Glenn Hepfer, Brooke J. Damon, Changcheng Shi, Jenny J. Yao, Matthew C. Coombs, Michael J. Kern, Tong Ye, Hai Yao

Content

Supplementary Figure 1-10

Supplementary Table 1-4

Supplementary Note 1-7

Supplementary References

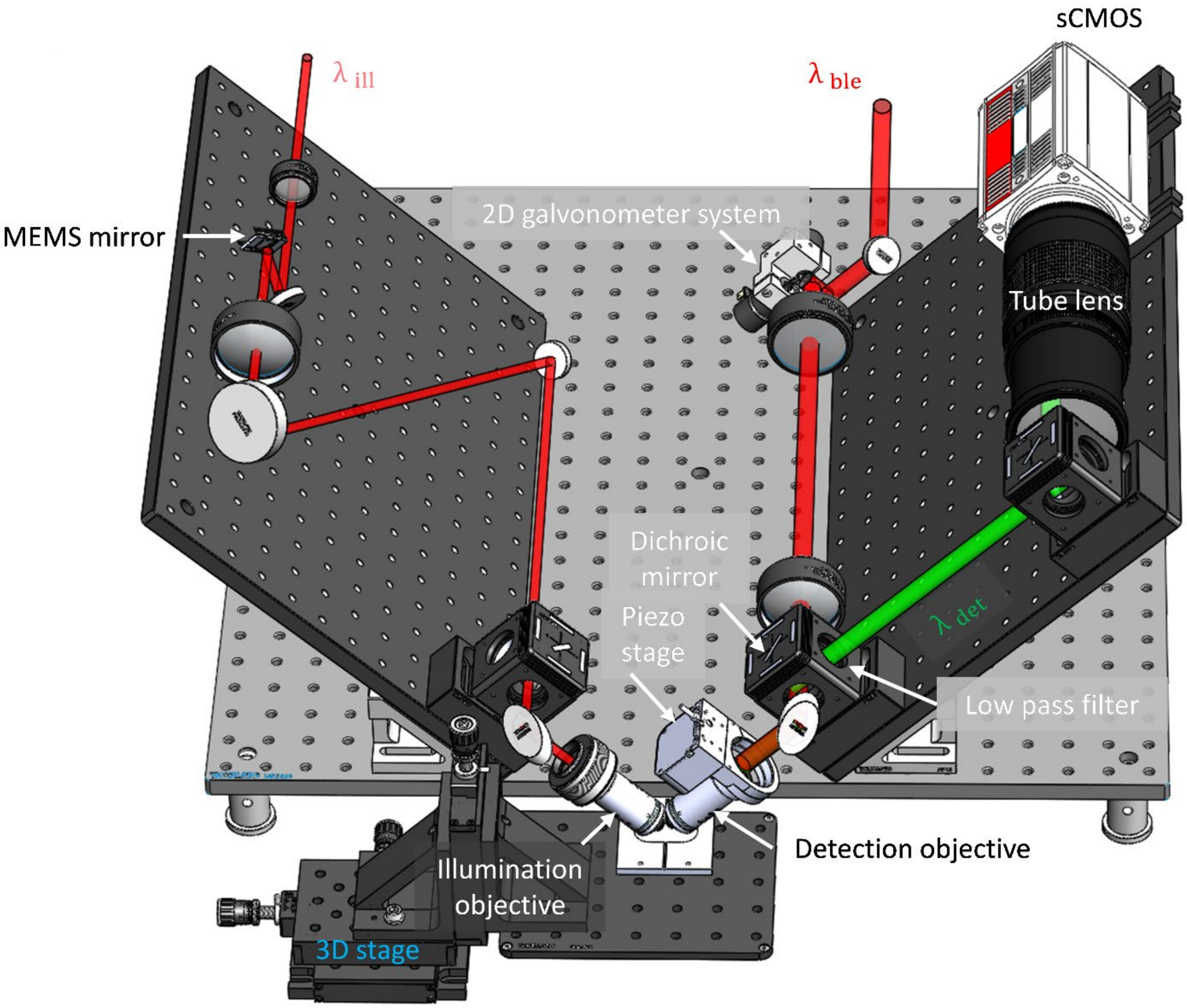

Supplementary Figure 1: LiFT-FRAP system layout. The LiFT-FRAP system is built on two breadboards tilted at 45° with respect to the surface of the air table. The light-sheet unit is on the left breadboard with the illumination laser path labeled in red. The bleaching volume generator and detection unit are on the right breadboard with the bleaching laser path labeled in red and detection path labeled in green. Major hardware components are labeled in white or black. Samples are anchored on a 3D stage (labeled in blue) that moves the samples in x, y and z directions. λ_{ill}, illumination laser; λ_{det}, detected emission fluorescence; λ_{ble}, bleaching laser.

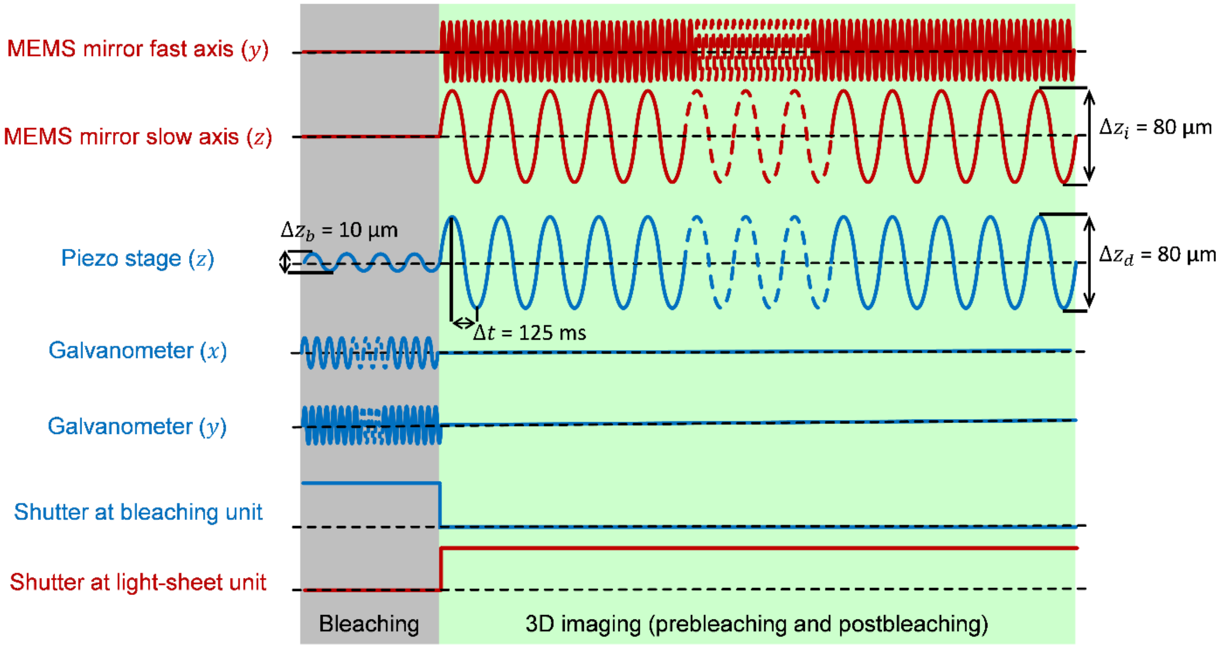

Supplementary Figure 2: Voltage signals that operate the LiFT-FRAP experiment. Each control signal uses different waveforms in the bleaching (grey area) and 3D imaging (green area) cycles. Control signals for components in the light-sheet unit are labeled in red while those for the bleaching volume generator and detection unit are labeled in blue. Horizontal dash lines labeled in black indicate the baseline for each signal. Dashed waveforms shown in the control signals of the MEMS mirror, piezo stage, and 2D galvanometer system indicate multiple repeats of the waveform that are not shown in this figure. During the bleaching cycle, the control signal for the MEMS mirror is parked at its center position with a constant voltage (baseline). Piezo stage is controlled by a sinusoidal waveform, leading to a 10 µm range of movement along z-axis (Δz_b). Two sinusoidal waveforms at frequencies of 50 Hz and 500 Hz control the scanning of 2D galvanometer system in x-axis and y-axis respectively. A high-level digital I/O signal opens the shutter in the bleaching volume generator while a low-level digital I/O signal closes the shutter in the light-sheet unit. During the 3D imaging cycle, the bleaching shutter is closed while the light-sheet shutter is opened. Two sinusoidal waveforms at frequencies of 1,280 Hz and 4 Hz drive the MEMS mirror to scan along y-axis and z-axis respectively. The range of movement of the illumination plane along z-axis (Δz_i) is 80 µm. A sinusoidal waveform at a frequency of 4 Hz controls the movement of piezo stage, leading an 80-µm range of movement along z-axis (Δz_d). It takes 125 ms (Δt) to complete the acquisition of a volumetric image. A voltage pulse initiates the image acquisition with the camera at the beginning of each 3D imaging cycle.

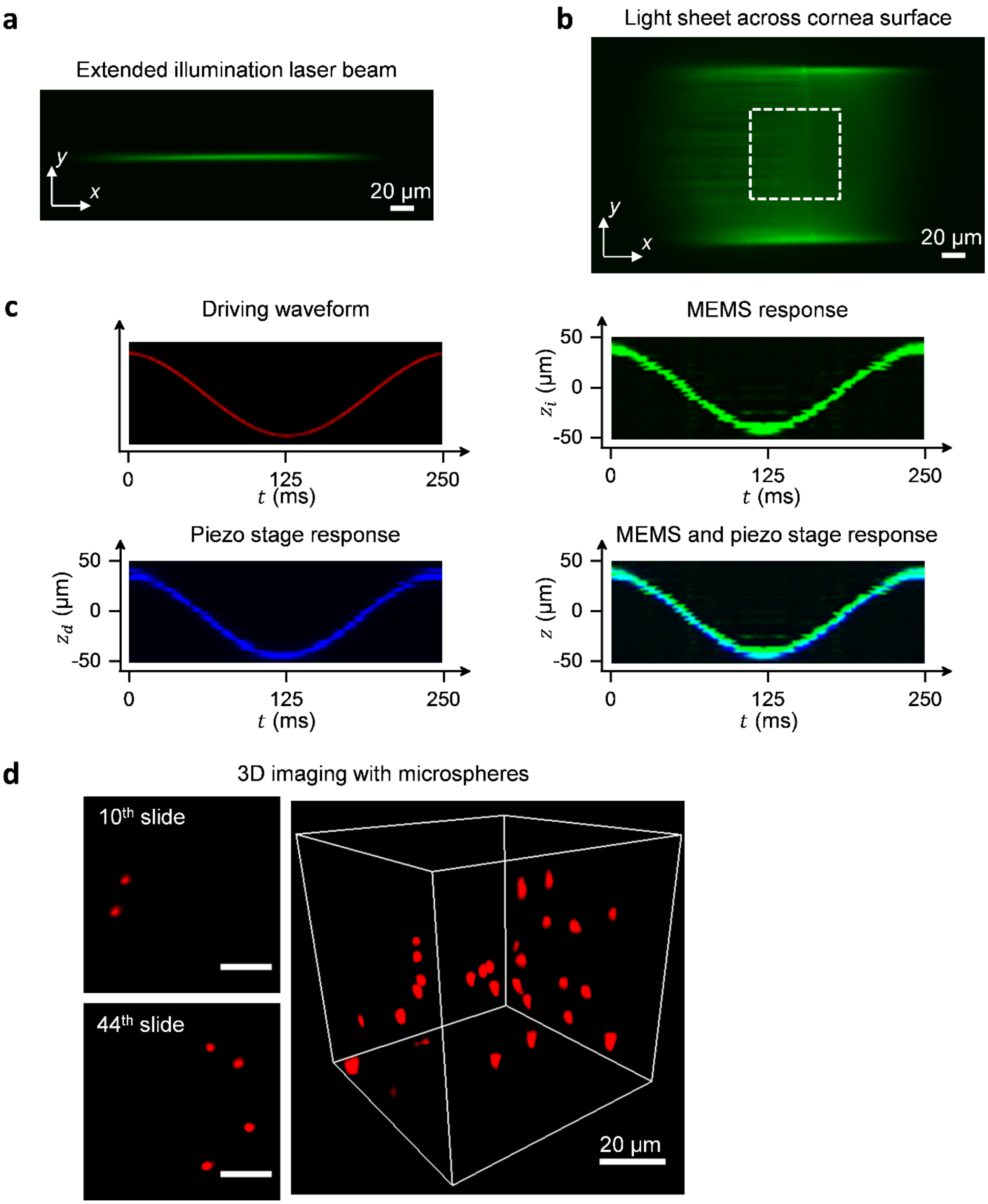

Supplementary Figure 3: LiFT-FRAP system 3D imaging performance. a Extended Gaussian beam (Experiments were repeated in 3 independent samples). The illumination laser was extended in the x direction, which was visualized in sodium fluorescein solutions. **b** Light sheet captured by sCMOS camera when intersecting the cornea surface (Experiments were repeated in 7 independent samples). Porcine cornea was stained by FD20 in situ. A visible,

vertical line in the middle indicates the interface between the cornea tissue (left side) and the staining solution (right side). The images used in LiFT-FRAP experiments is cropped by the dashed box in white. **c** Synchronization performance for the movement of the light-sheet illumination plane and the detection plane during 3D imaging (Experiments were repeated in 2 independent samples). The movement of piezo stage and the scanning of MEMS mirror along the slow axis was controlled by a similar sinusoidal waveform (red). The dynamic response (Supplementary Note 3) of piezo stage (blue) and MEMS mirror (green) were measured. Merged images of the dynamic response of MEMS mirror and piezo stage is shown in the bottom right. **d** 3D imaging of fluorescent microspheres of 1.75 μm in diameter in 1% agarose gel (Experiments were repeated in 5 independent samples). Left, individual 2D images; Right, the 3D reconstruction. All scale bars in this figure represent 20 μm.

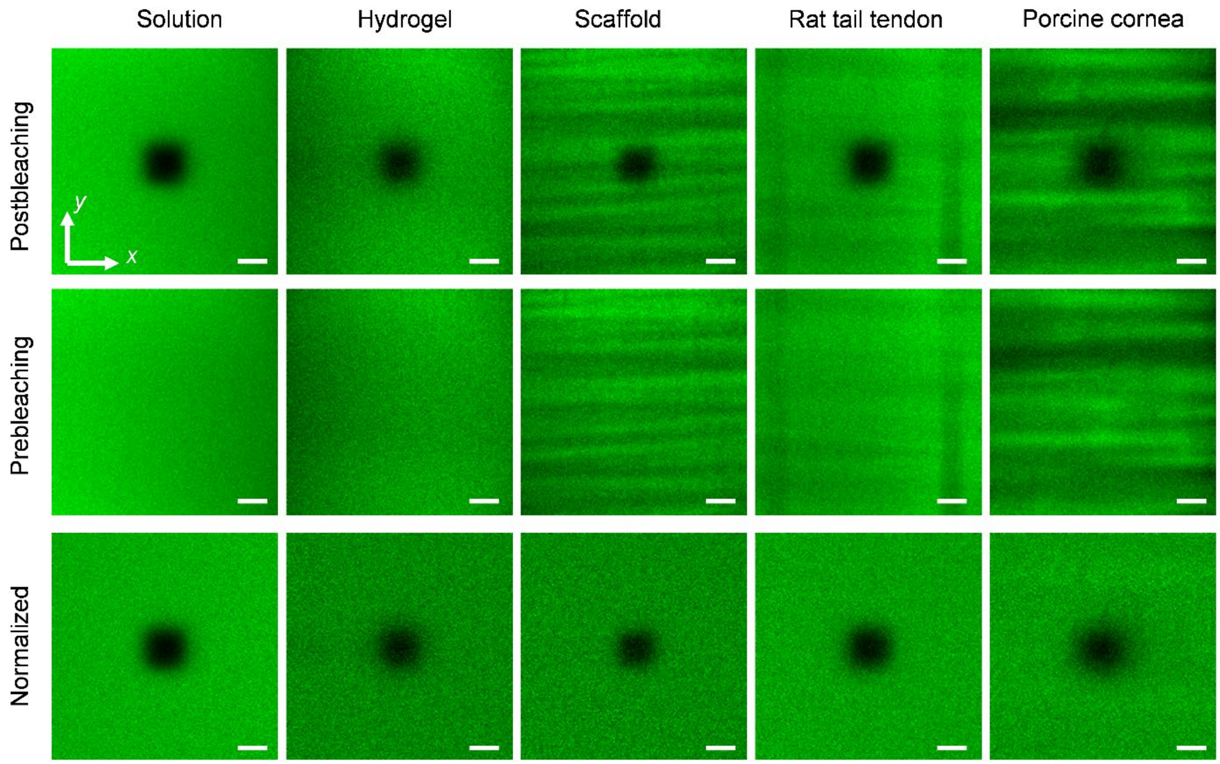

Supplementary Figure 4: LiFT-FRAP image normalization. The background of postbleaching images are not uniform due to possible non-uniform dye distribution within the sample, uneven illumination, and stripe artifacts caused by refractive index mismatching. Images with uniform background are obtained by normalizing the postbleaching images with prebleaching images (Supplementary Note 4**)**. Scale bar, 10 μm. Experiments were repeated in at least 5 independent samples.

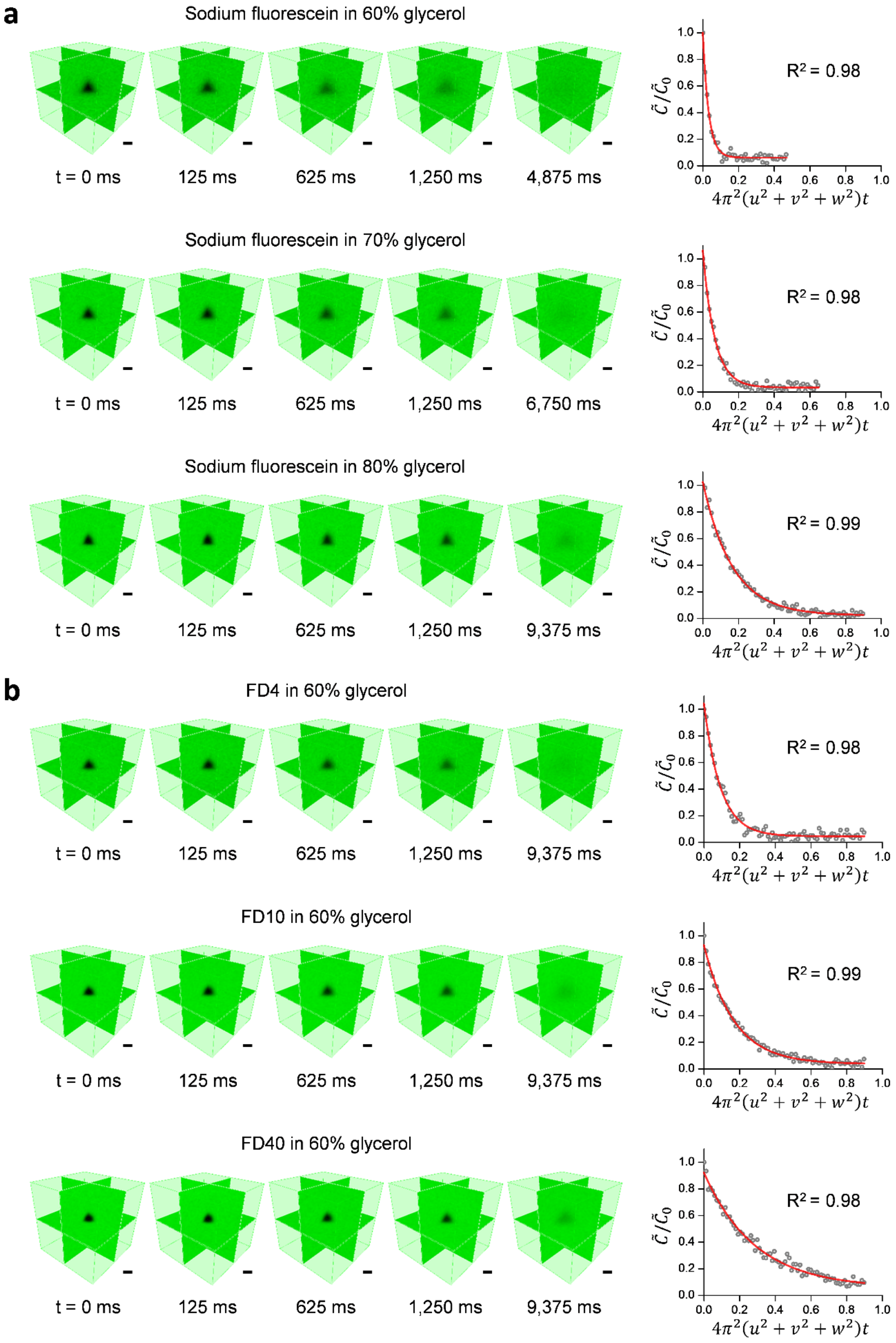
a
Sodium fluorescein in 60% glycerol
t = 0 ms
125 ms
625 ms
1,250 ms
4,875 ms
R² = 0.98
Sodium fluorescein in 70% glycerol
t = 0 ms
125 ms
625 ms
1,250 ms
6,750 ms
R² = 0.98
Sodium fluorescein in 80% glycerol
t = 0 ms
125 ms
625 ms
1,250 ms
9,375 ms
R² = 0.99
b
FD4 in 60% glycerol
t = 0 ms
125 ms
625 ms
1,250 ms
9,375 ms
R² = 0.98
FD10 in 60% glycerol
t = 0 ms
125 ms
625 ms
1,250 ms
9,375 ms
R² = 0.99
FD40 in 60% glycerol
t = 0 ms
125 ms
625 ms
1,250 ms
9,375 ms
R² = 0.98
$\tilde{C}/\tilde{C}_0$
$4\pi^2(u^2 + v^2 + w^2)t$

Supplementary Figure 5: LiFT-FRAP experiment in solutions. **a** Typical time series of LiFT-FRAP 3D normalized images of sodium fluorescein in glycerol solutions with different viscosities (glycerol concentration: 60%, 70%, and 80%, v/v) and corresponding curve fitting results. **b** Typical time series of LiFT-FRAP 3D normalized images of FD molecules (FD4, FD10, and FD40) in 60% (v/v) glycerol solutions and corresponding curve fitting results. Grey circles are the normalized amplitudes of the solute concentration in spatial frequency domain ($\tilde{C}/\tilde{C}_0$) at different time points. Red lines show the corresponding fitting curves. The unit of x axis is μm^{-2} s. All scale bars in this figure represent 10 μm.

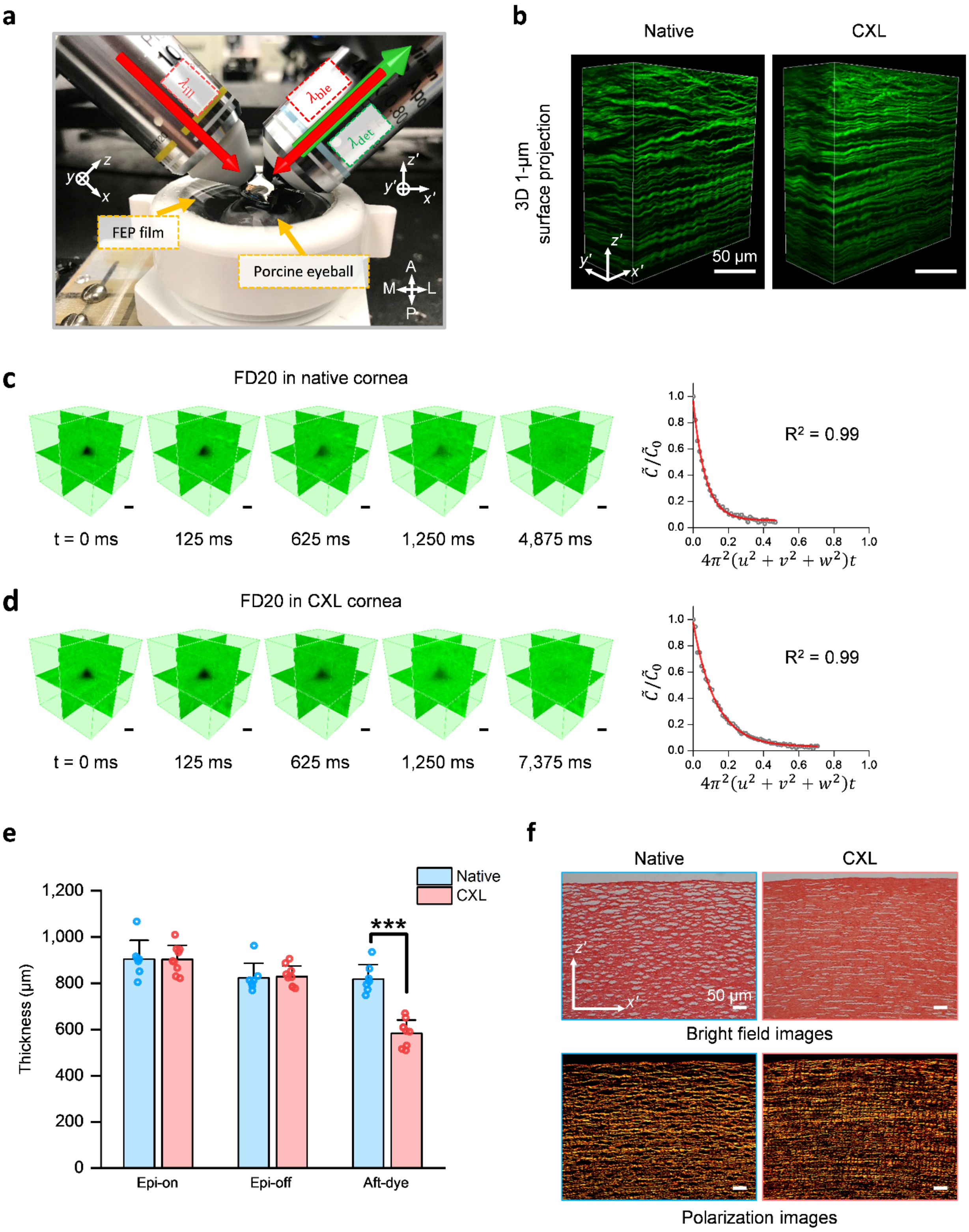

Supplementary Figure 6: LiFT-FRAP experiment in cornea tissues. a Experimental setup for cornea experiments. LiFT-FRAP coordinate system is labeled as x-y-z while cornea coordinate

system is labeled as x'-y'-z'. A, anterior; P, posterior; M, medial; L, lateral: indicate the orientation of porcine cornea. λ_{ill}, illumination laser; λ_{det}, detected emission fluorescence; λ_{ble}, bleaching laser; FEP film, fluorinated ethylene propylene film. **b** Representative 3D SHG images of native and CXL corneas (Experiments were repeated in 5 independent samples). Scale bar, 50 µm. **c** Typical time series of LiFT-FRAP 3D normalized images of FD20 in native porcine corneas and corresponding curve fitting results. Scale bar, 10 µm. **d** Typical time series of LiFT-FRAP 3D normalized images of FD20 in CXL porcine corneas and corresponding curve fitting results. Scale bar, 10 µm. **e** Cornea thickness measured in native (n = 7 independent corneas) and CXL (n = 9 independent corneas) corneas. Epi-on, epithelial-on cornea thickness; Epi-off, epithelial-off cornea thickness; Aft-dye, after-dye cornea thickness. *** $p<0.0001$, two-sided t test. Data depict mean ± standard deviation. **f** Representative bright field and polarization images of picrosirius red stained sections of native and CXL porcine corneas (Experiments were repeated in 3 independent samples). Scale bar, 50 µm.

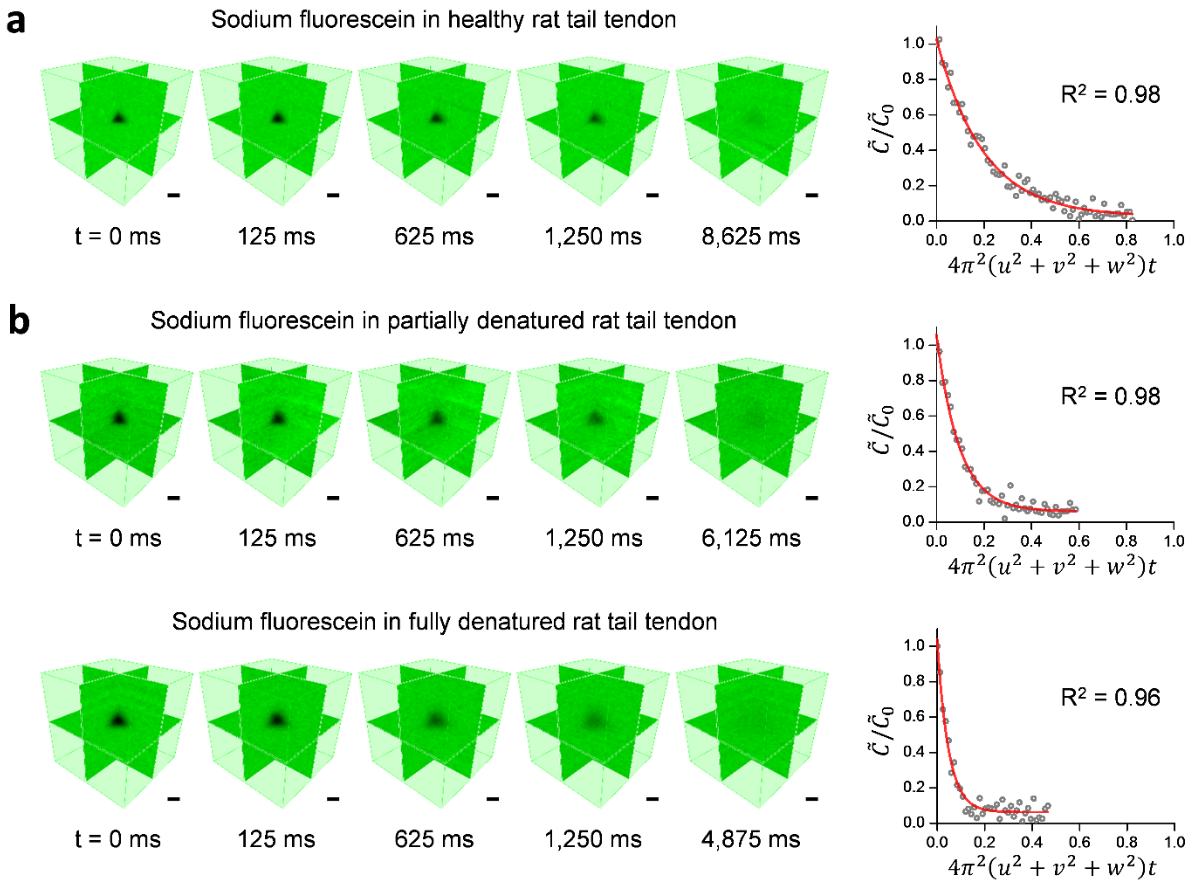

Supplementary Figure 7: LiFT-FRAP experiment in tendon tissues. a Typical time series of LiFT-FRAP 3D normalized images of sodium fluorescein in healthy rat tail tendons and corresponding curve fitting results. **b** Typical time series of LiFT-FRAP 3D normalized images of sodium fluorescein in thermally treated rat tail tendons (partially denatured and fully denatured) and corresponding curve fitting results. All scale bars in this figure represent 10 µm.

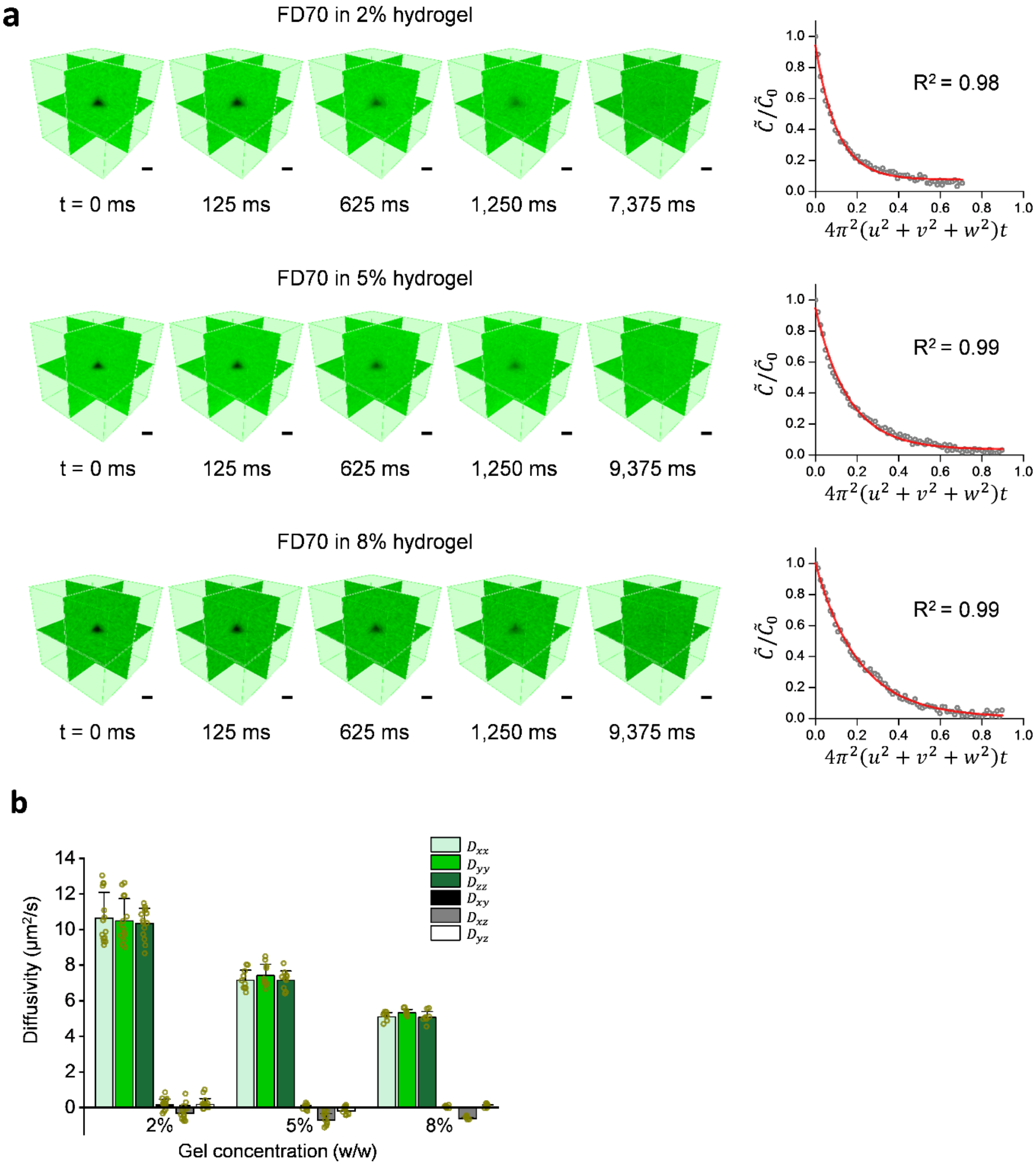

Supplementary Figure 8: LiFT-FRAP experiment in gelation hydrogels. a Typical time series of LiFT-FRAP 3D normalized images of FD70 in gelatin hydrogels with different gelatin concentrations (2%, 5%, and 8%, w/w) and corresponding curve fitting results. Scale bar, 10 µm. **b** 3D diffusion tensor results of FD70 in 2%, 5%, and 8% gelatin hydrogels (n = 14 for 2%, n = 10 for 5%, and n = 8 for 8%). Data depict mean ± standard deviation.

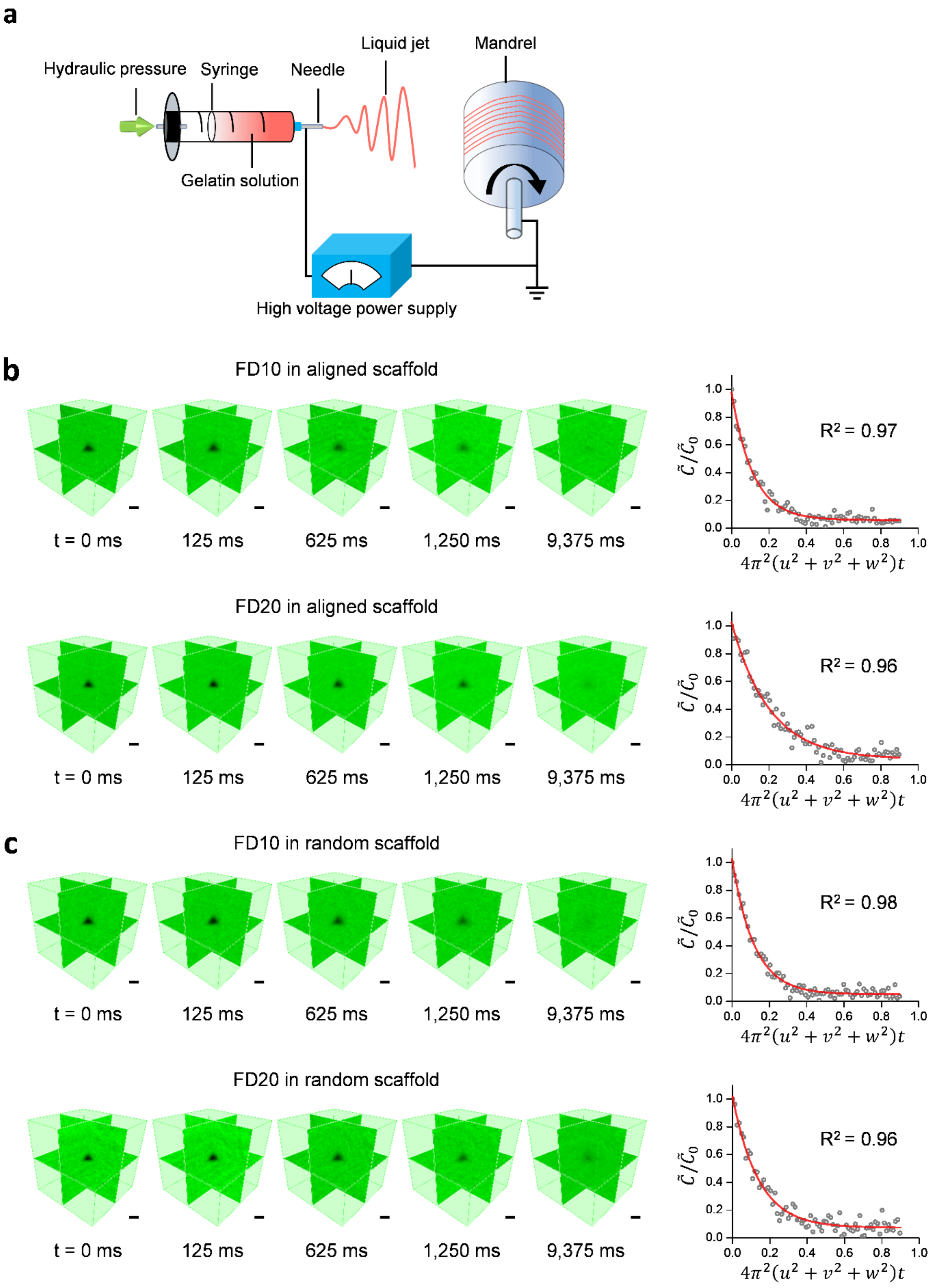
a
Hydraulic pressure
Syringe
Needle
Liquid jet
Mandrel
Gelatin solution
High voltage power supply
b
FD10 in aligned scaffold
t = 0 ms
125 ms
625 ms
1,250 ms
9,375 ms
R² = 0.97
FD20 in aligned scaffold
t = 0 ms
125 ms
625 ms
1,250 ms
9,375 ms
R² = 0.96
c
FD10 in random scaffold
t = 0 ms
125 ms
625 ms
1,250 ms
9,375 ms
R² = 0.98
FD20 in random scaffold
t = 0 ms
125 ms
625 ms
1,250 ms
9,375 ms
R² = 0.96
$\tilde{C}/\tilde{C}_0$
$4\pi^2(u^2+v^2+w^2)t$

Supplementary Figure 9: LiFT-FRAP experiment in fiber-based scaffolds. a Electrospinning setup for fabricating fiber-based scaffolds. The mandrel is rotating during electrospinning for fiber-aligned scaffold fabrication but remains stationary for random fiber scaffold collection. **b** Typical time series of LiFT-FRAP 3D normalized images of FD10 and FD20 in fiber-aligned scaffolds and corresponding curve fitting results. **c** Typical time series of LiFT-FRAP 3D normalized images of FD10 and FD20 in random fiber scaffolds and corresponding curve fitting results. All scale bars in this figure represent 10 μm.

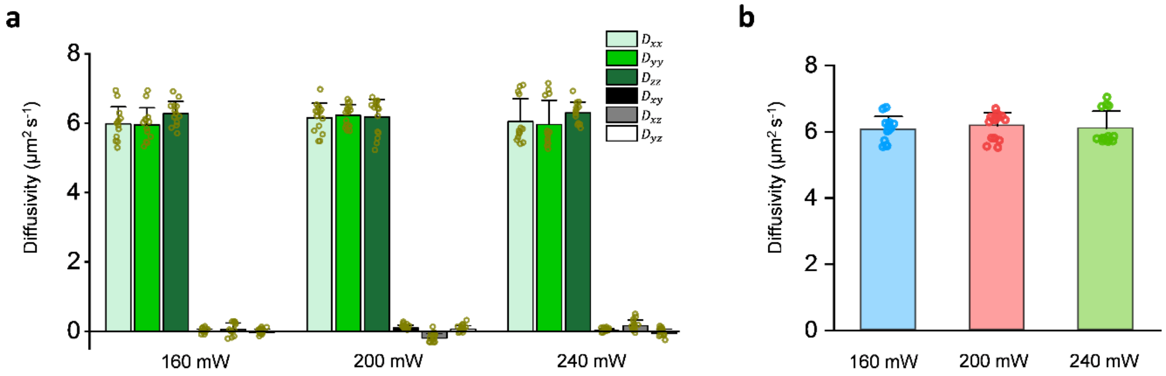

Supplementary Figure 10: LiFT-FRAP measurement at different bleaching laser powers. a 3D diffusion tensors of sodium fluorescein in 80% glycerol solution with different bleaching laser powers: 160 mW (n=13 independent measurements), 200 mW (n=15 independent measurements), 240 mW (n=13 independent measurements) (laser power measured right after the objective). LiFT-FRAP experiments were performed on sodium fluorescein in 80% glycerol (v/v) solution with different bleaching laser power settings to investigate the possible local heating effect by the bleaching laser illumination on the diffusion measurement. The results showed that the diffusion of sodium fluorescein remains isotropic for all three groups, and the diffusion tensors do not change with the alteration of the bleaching laser. **b** Average diffusivity of sodium fluorescein in 80% glycerol solution. (n=13 independent measurements for 160 mW; n=15 independent measurements for 200 mW; n=13 independent measurements for 240 mW) The average diffusivities are the average values of the diagonal components of the 3D diffusion tensor. No significant diffusivity differences were found among the three groups ($p = 0.7$, one-way ANOVA), indicating that the influence of local heating during the bleaching process on molecular diffusion is negligible. All data depict mean ± standard deviation.

Supplementary Table 1. Molecular diffusivities in biological systems.

Molecule	Molecular weight (Da)	Biological system	Diffusivity ($\mu m^2\ s^{-1}$)
Glucose	180	Human cartilage endplate	19.1-34.4 [1]
Pyronin Y	303	Bovine flexor tendon	27.9-36.8 [2]
Fluorescein isothiocyanate	389	Breast cancer microtissue	21.0 [3]
VEGF-biomimetic peptide	3,810	Hydrogel	15.2 [4]
Insulin-like growth factor	7,600	Bovine articular cartilage	26 [5]
Myoglobin	17,000	Human cornea	5.5 [6]
Green fluorescence protein	27,000	Zebrafish embryo	36 [7]
Bovine serum albumin	66,000	Dextran scaffold	15 [8]
Human transferrin	80,000	Rat cortex	14 [9]
IgG	150,000	Human soft tissue sarcoma	9.6 [10]
FD167*	167,000	Bovine cystic fibrosis mucus	6.4 [11]

***** FD**:** fluorescein isothiocyanate-conjugated dextran

Supplementary Table 2. Theoretical diffusivities of sodium fluorescein at different glycerol solutions. Theoretical diffusivities were calculated based on the Stokes-Einstein equation (Supplementary Note 6). The Stokes radius of sodium fluorescein is 0.45 nm. Viscosity data at room temperature (20°C) was retrieved from literature [12]. Theoretical prediction curve shown in Fig. 3c is an exponential fit of the theoretical diffusivities listed below.

Glycerol concentration % (v/v)	Viscosity ($\times 10^{-3}$ N m^{-2} s)	Theoretical diffusivities (μm^2 s^{-1})
54.3	10.8	44.137
59.6	15.2	31.361
61.7	17.7	26.931
64.9	22.5	21.186
70.4	35.5	13.428
76.0	60.1	7.932
81.8	109	4.373

Supplementary Table 3. Theoretical diffusivities of fluorescence molecules with various molecule sizes in solutions. Theoretical diffusivities were calculated based on the Stokes-Einstein equation (Supplementary Note 6). Viscosity of $\eta = 15.2 \times 10^{-3}$ N m^{-2} s was used for the 60% glycerol solution at room temperature (20°C). Theoretical prediction curve shown in Fig. 3e is a linear fit of the theoretical diffusivities listed below in the log-log graph.

Molecules	Manufacturers	Stokes radius (nm)	Solution viscosity ($\times 10^{-3}$ N m^{-2} s)	Theoretical diffusivities (μm^2 s^{-1})
Sodium fluorescein	Sigma-Aldrich	0.45	15.2	31.361
FD4	Sigma-Aldrich	1.4	15.2	10.080
FD10	Sigma-Aldrich	2.3	15.2	6.136
FD40	Sigma-Aldrich	4.5	15.2	3.136

Supplementary Table 4. LiFT-FRAP experiment parameters.

Sample type	Measurement depth (μm)	Postbleaching image acquisition time (ms)	Light-sheet laser power (mW)*	Bleaching laser power (mW)*
Solution	150	9,375	135 – 180	160
Porcine cornea	150	7,375	225 – 270	280
Rat tail tendon	150	8,625	225 – 270	120 – 160
Hydrogel	150	9,375	225	80
Fiber-based scaffold	100	9,375	315	120 – 160

* Laser power was measured right after the objective.

Supplementary Notes

Supplementary Note 1: Modeling 3D molecular dynamics and concentration distributions

To characterize the impact of isotropic and anisotropic diffusion on 3D molecular dynamics and concentration distributions, we developed a computational model to simulate the process of molecules diffusing out from a central source, such as cells, within a 3D domain. The spherical subdomain at the center of the cubic domain had a diameter of 10 μm and was assigned a constant concentration as the molecular source. The 3D cubic domain with side length 40 μm had a no-flux boundary condition, and its initial concentration was set to zero. The molecular concentration profile was governed by the diffusion equation (Fick's second law) [13]:

$$\frac{\partial}{\partial t}C(x,y,z,t) = \nabla \cdot \{\mathbf{D}[\nabla C(x,y,z,t)]\} \tag{1}$$

where (x,y,z) is the spatial position, t is time and $\mathbf{D} = \begin{bmatrix} D_{xx} & D_{xy} & D_{xz} \\ D_{xy} & D_{yy} & D_{yz} \\ D_{xz} & D_{yz} & D_{zz} \end{bmatrix}$ is the 3D diffusion tensor of the diffusing molecules. $C(x,y,z,t)$ is the concentration of diffusing molecules. To simplify the simulation, we assumed that diffusion occurs only along the principal directions, where $\mathbf{D} = \begin{bmatrix} D_{0xx} & 0 & 0 \\ 0 & D_{0yy} & 0 \\ 0 & 0 & D_{0zz} \end{bmatrix}$. Four 3D diffusion tensors, with an identical average diffusivity of 10 $\mu m^2/s$ but different diffusion anisotropies (Fig. 1c), were simulated to reveal the differences between isotropic diffusion and anisotropic diffusion. To evaluate the modeling results, all concentrations were normalized to the source subdomain value, and magnitudes of all diffusion fluxes $\mathbf{J} = -\mathbf{D}[\nabla C(x,y,z,t)]$ were normalized to the maximum magnitude of diffusion flux among all models at each time point. The normalized concentration distribution profile and diffusion flux among different models were compared in three orthogonal planes at each time step. In addition, normalized concentration profiles from the source to the edge of the cubic domain were compared along each axis for each model at each time step. The simulation results were plotted with the Tecplot 360 (Tecplot, Bellevue, WA).

To demonstrate the impact of inaccurate 2D measurement on tissue biochemical environment, we simulated the 3D molecular diffusion in cornea tissues. A spherical geometry of human cornea was assumed [14], as shown in Fig. 5a. The radius of anterior cornea was set to be 7.48 mm, and cornea thickness was 500 μm. We assigned a constant concentration to a portion of anterior cornea (an area with a radius of 1 mm) to simulate the delivery of biomolecules, such as drugs, from anterior cornea to posterior cornea. All the boundaries were assigned as a no-flux boundary. The diffusion of FD20 in native and CXL corneas measured through both LiFT-FRAP and 2D FRAP experiments were used as the diffusion input. To compare the differences among the four simulations, we plot the normalized concentration profiles along the cornea axis, as well as the normalized molecular concentration at the posterior cornea apex.

All models were solved using finite element analysis software (COMSOL Multiphysics 5.4, COMSOL Inc., Burlington, MA).

Supplementary Note 2: LiFT-FRAP system control

The voltage control signals of the LiFT-FRAP system operation were provided by two DAQ boards (PCI-6115 and PCI-6713, National Instruments, Austin, TX) synchronized with an

RTSI bus cable. A customized software (available upon request) written in LabVIEW 2014 32-bit (National instruments, Austin, TX) managed the timing and all waveforms of control signals applied to the MEMS mirror, piezo stage, shutters, 2D galvanometer system and sCMOS camera (Supplementary Fig. 2). Each control signal uses different waveforms in the bleaching and 3D imaging cycles. During the bleaching period, the control voltage for the MEMS mirror was set at a fixed level, leading to none scanning of the MEMS mirror. The piezo stage instead was driven by a sinusoidal waveform with the frequency of 4 Hz, which allowed the scanning of the focused bleaching laser along the z-axis, and the amplitude was set to have a 10-μm range of movement along the z-axis. To create a 3D bleaching volume, the 2D galvanometer system was controlled by two sinusoidal waveforms with the frequency of 50 Hz and 500 Hz to concurrently scan the bleaching laser in the x-axis and y-axis, respectively. The shutter on the bleaching beam path was opened when a high-level digital input/output (I/O) was applied to the shutter controller. The shutter on the light-sheet path was kept close while a low-level digital I/O signal was applied. During 3D imaging, including prebleaching and postbleaching, two sinusoidal waveforms were used to control the scanning of MEMS mirror. The fast axis scanning of the MEMS mirror was controlled by a waveform with the frequency of 1,280 Hz, which allowed the scanning of an extended Gaussian beam (Supplementary Fig. 3a) along the y-axis, generating the light sheet (Supplementary Fig. 3b). The slow axis scanning of the MEMS mirror instead was input with a waveform with the frequency of 4 Hz, which permitted the continuous 3D illumination. To acquire 3D in-focus prebleaching and postbleaching images, the movement of the detection plane and illumination plane needs to be synchronized during 3D imaging. We therefore applied a sinusoidal waveform with the same frequency (4 Hz) as the control signal used for scanning the MEMS mirror in the slow axis to drive the movement of piezo stage. The amplitude of control signals for both MEMES and piezo stage were adjusted to achieve 80-μm range of movement for the illumination plane and detection plane along z-axis, respectively. The shutter at the volume bleaching generator was closed with a low-level digital I/O input signal while the shutter at the light-sheet unit was open with a high-level digital I/O input signal. The sCMOS camera was triggered by a voltage pulse to start taking 2D images at 624 frames per second once the bleaching process was finished. The images were streamed and stored in a high-speed M.2 solid state hard drive for post processing.

Supplementary Note 3: LiFT-FRAP system 3D imaging performance

The extended Gaussian beam was visualized by illuminating sodium fluorescein in 80% glycerol solutions without the scanning of MEMS mirror and captured with the sCMOS camera (Supplementary Fig. 3a). The full width at the half maximum of the extended Gaussian beam was 4.8 ± 0.3 μm, which was significantly larger than the imaging depth (about 0.5 μm) of the detection objective. The large Raleigh length of the Gaussian beam ensured the field of view to be sufficient for the 3D FRAP experiment. The light sheet was captured by illuminating the in-situ porcine corneas stained with 20 kDa fluorescein isothiocyanate-dextran (FD) at a region close to the tissue surface (Supplementary Fig. 3b). In this case, MEMS mirror only scanned along the y-axis to generate the light sheet.

In the LiFT-FRAP system, the illumination plane and detection plane were independently scanned over a distance of 80 μm to achieve the 3D imaging. The overlap of the illumination plane and detection plane during 3D imaging is critical. The synchronization of the movement of both planes were ensured by tuning the waveforms applied to the MEMS mirror and piezo stage. To test the movement synchronization of the illumination plane and detection plane during 3D

imaging, we measured the dynamic behavior of MEMS mirror and piezo stage in response to the control signal [15]. A 1% agarose gel mixed with fluorescent microsphere (1.75 μm in diameter, Polysciences Inc., Warrington, PA) was prepared for this test. In brief, a constant signal was used to control the piezo stage, which located the detection plane at position $z_d = z_0$. The control signals used for MEMS mirror in LiFT-FRAP experiments (Supplementary Fig. 2 and Supplementary Fig. 3c) were applied to the MEMS mirror to continuously scan the illumination laser in a 3D volume. The sCMOS camera recorded the fluorescent signal from the observed region (76 μm × 76 μm) of the detection plane z_0 at 624 frames per second. When the position of light-sheet illumination plane z_i was overlapped with the z_d, the emitted fluorescence signal from the fluorescent microsphere recorded at that time t_0 was the brightest. As the position of the detection plane was predefined, $z_d = z_0$, the position of this illumination plane with maximum fluorescence at time t_0 could be determined, $z_i = z_0$. Following the same procedure with a series of the detection plane position ($z_d = z_0$, $z_1 \ldots z_{99}$), a series of detection plane position z_i can be determined. Each z_i was corresponding to a frame with maximum fluorescence captured at specific time t. Therefore, the dynamic response of MEMS mirror can be reconstructed (Supplementary Fig. 3c). In this test, z_d was set in the range of [-50, 50], which was larger than the range of movement of the illumination plane, 80 μm. The results showed that fluorescent signal followed the driving waveform and was detected only within the rage of [-40, 40], indicating that the MEMS mirror dynamic response was well controlled. Similarly, to determine the dynamic response of the piezo stage during 3D imaging, the illumination plane was fixed at a given location while the piezo stage was driven to scan the detection plane in the z direction using the sinusoidal signal (Supplementary Fig. 2 and Supplementary Fig. 3c). Similar to the procedure for determination of the dynamic response for MEMS mirror, the position of z_d at time t can also be determined (Supplementary Fig. 3c). The results also showed the piezo stage was well controlled to move in the range of 80 μm. The dynamic responses of the MEMS mirror and piezo stage were well overlapped, indicating good synchronization of illumination plane and detection plane during 3D imaging (Supplementary Fig. 3c).

When the illumination plane exactly overlaps with the focal plane of the detection objective, the image captured by the sCMOS camera is in-focus. Otherwise the image will be blurred. To evaluate the performance of LiFT-FRAP system for 3D imaging, we imaged a fluorescent microsphere (1.75 μm in diameter) in 1% agarose gel. The results showed high contrast 2D images of the microsphere (Supplementary Fig. 3d) with successful 3D reconstruction of beads distributed in the gel (Supplementary Fig. 3d).

Supplementary Note 4: Imaging normalization

In LiFT-FRAP experiments, possible non-uniform distribution of the fluorescent molecules in the samples, uneven illumination, and stripe artifacts caused by the mismatching of the refractive indices along the light sheet propagation direction [16] will cause a non-uniform image background (Supplementary Fig. 4). To obtain uniform background images, prebleaching images were recorded at the start of each LiFT-FRAP experiment. Therefore, each postbleaching image had its own corresponding prebleaching image. By normalizing the postbleaching image with the prebleaching image [17],

$$I_n(x, y, t) = \frac{I_{\text{post}}(x, y, t)}{I_{\text{pre}}(x, y, t)} \quad (2)$$

where $I_n(x,y,t)$ is the normalized intensity at pixel (x,y) at time t, $I_{\text{pre}}(x,y,t)$ and $I_{\text{post}}(x,y,t)$ are the intensities at pixel (x,y) at time t in the prebleaching image and postbleaching image, respectively. A uniform background around the bleaching area was obtained (Supplementary Fig. 4).

Supplementary Note 5: 3D Fourier transform FRAP theory

Data analysis of LiFT-FRAP is based on our previously published 3D Fourier transform FRAP theory (Fig. 2b) [18]. In LiFT-FRAP experiment, 3D fluorescence recovery process after photobleaching is governed by Fick's second law shown in equation (1). A 3D Fourier transformation can be performed to solve equation (1) with the boundary condition that $C(x,y,z,t)$ is constant as $(x,y,z) \to \pm\infty$ [18].

$$\frac{\tilde{C}(u,v,w,t)}{\tilde{C}(u,v,w,0)} = \exp\left[-4\pi^2(u^2+v^2+w^2)D(u,v,w)t\right] \tag{3}$$

where $\tilde{C}(u,v,w,t)$ is defined as $\tilde{C}(u,v,w,t) = \int_{-\infty}^{\infty}\int_{-\infty}^{\infty}\int_{-\infty}^{\infty} C(x,y,z,t)e^{-2\pi i(ux+vy+wz)}\,dxdydz$ and $D(u,v,w)$ is defined as:

$$D(u,v,w) = \frac{u^2D_{xx} + v^2D_{yy} + w^2D_{zz} + 2uvD_{xy} + 2uwD_{xz} + 2vwD_{yz}}{u^2+v^2+w^2} \tag{4}$$

where u, v, and w are the spatial frequencies.

Equation (3) is the basis for the LiFT-FRAP analysis. The value of $D(u,v,w)$ in the spatial frequency domain can be calculated by fitting equation (3) to the experimental data (Fig. 2b). To determine individual diffusion tensor component, the average value of $D(u,v,w)$ is calculated over a spherical surface in spatial frequency domain:

$$D_{\alpha_1,\alpha_2,\beta_1,\beta_2} = \frac{\iint D(u,v,w)dS}{\iint dS} = \frac{\int_{\beta_1}^{\beta_2}\int_{\alpha_1}^{\alpha_2} D(\phi,\theta)\sin\theta\, d\phi d\theta}{[(\alpha_2-\alpha_1)(\cos\beta_1-\cos\beta_2)]} \tag{5}$$

where

$$\begin{aligned} D(\phi,\theta) = {} & (\sin\theta\cos\phi)^2 D_{xx} + (\sin\theta\sin\phi)^2 D_{yy} + (\cos\theta)^2 D_{zz} \\ & + (\sin\theta)^2\sin 2\phi\, D_{xy} + \sin 2\theta\cos\phi\, D_{xz} + \sin 2\theta\sin\phi\, D_{yz} \end{aligned} \tag{6}$$

is the expression of $D(u,v,w)$ in the spherical coordinate, where ϕ is the azimuthal angle ranging from α_1 to α_2, θ is polar angle ranging from β_1 to β_2. By averaging over different spherical surfaces, each component of the 3D diffusion tensor is determined (Fig. 2b) [18]:

$$
\begin{cases}
D_{xy} = \frac{3\pi}{4}\left(D_{0,\pi/2,0,\pi} - D_{0,\pi,0,\pi}\right) \\
D_{yz} = \frac{3\pi}{4}\left(D_{0,\pi,0,\pi/2} - D_{0,\pi,0,\pi}\right) \\
D_{xz} = \frac{3\pi}{4}\left(D_{0,\pi/2,0,\pi/2} + D_{0,\pi,0,\pi} - D_{0,\pi/2,0,\pi} - D_{0,\pi,0,\pi/2}\right) \\
D_{xx} = (2\sqrt{2}+2)D_{0,\pi,0,3\pi/4} - 2\sqrt{2}D_{0,\pi,0,\pi} - D_{0,\pi,0,\pi/2} + \frac{3\pi}{4}\left(D_{0,\pi/4,0,\pi} - D_{0,\pi/2,0,\pi}\right) \\
D_{yy} = (2\sqrt{2}+2)D_{0,\pi,0,3\pi/4} - 2\sqrt{2}D_{0,\pi,0,\pi} - D_{0,\pi,0,\pi/2} - \frac{3\pi}{4}\left(D_{0,\pi/4,0,\pi} - D_{0,\pi/2,0,\pi}\right) \\
D_{zz} = \left(4\sqrt{2}+3\right)D_{0,\pi,0,\pi} + 2D_{0,\pi,0,\pi/2} - (4\sqrt{2}+4)D_{0,\pi,0,3\pi/4}
\end{cases} \tag{7}
$$

Supplementary Note 6: Theoretical diffusivity calculation

The theoretical diffusivities of sodium fluorescein and FD molecules in glycerol solutions were calculated from the Stokes-Einstein equation [19]:

$$D = \frac{k_B T}{6\pi\eta r} \tag{8}$$

Where k_B = 1.38 × 10^{-23} m^2 kg s^{-2} K^{-1} is the Boltzmann's constant, T = 293 K is the room temperature, η is the solution viscosity, and r is the Stokes radius of the diffusing molecules. Solution viscosity and Stokes radius were collected from the literature or each manufacturers' website (Supplementary Table 2, 3).

Supplementary Note 7: Diffusion matrix rotation

In the LiFT-FRAP experiment with porcine corneas, the calculated 3D diffusion tensor **D** was rotated 45° counterclockwise along the y-axis (θ = 45°). The rotation matrix **R** was defined as,

$$\mathbf{R} = \begin{bmatrix} \cos\theta & 0 & -\sin\theta \\ 0 & 1 & 0 \\ \sin\theta & 0 & \cos\theta \end{bmatrix} \tag{9}$$

A new 3D diffusion tensor $\mathbf{D}' = \begin{bmatrix} D'_{xx} & D'_{xy} & D'_{xz} \\ D'_{xy} & D'_{yy} & D'_{yz} \\ D'_{xz} & D'_{yz} & D'_{zz} \end{bmatrix}$ can be derived by $\mathbf{D}' = \mathbf{R}^{\mathrm{T}}\mathbf{D}\mathbf{R}$, where $\mathbf{R}^{\mathrm{T}}$ is the transpose of **R**. After the matrix rotation, D'_{zz} is the diffusion in the anteroposterior direction (perpendicular to the lamellae). D'_{xx} and D'_{yy} instead are the diffusions in the mediolateral and superoinferior directions (parallel to lamellae structure), respectively.

Supplementary References

1 Wu, Y. *et al.* Region and strain-dependent diffusivities of glucose and lactate in healthy human cartilage endplate. *J Biomech* **49**, 2756-2762 (2016).

2 Hunckler, M. D., Tilley, J. M. & Roeder, R. K. Molecular transport in collagenous tissues measured by gel electrophoresis. *J Biomech* **48**, 4087-4092 (2015).

3 Brancato, V. *et al.* 3D breast cancer microtissue reveals the role of tumor microenvironment on the transport and efficacy of free-doxorubicin in vitro. *Acta Biomater* **75**, 200-212 (2018).

4 Mulyasasmita, W., Cai, L., Hori, Y. & Heilshorn, S. C. Avidity-controlled delivery of angiogenic peptides from injectable molecular-recognition hydrogels. *Tissue Eng Part A* **20**, 2102-2114 (2014).

5 Garcia, A. M. *et al.* Transport and binding of insulin-like growth factor I through articular cartilage. *Arch Biochem Biophys* **415**, 69-79 (2003).

6 Charalel, R. A. *et al.* Diffusion of protein through the human cornea. *Ophthalmic Res* **48**, 50-55 (2012).

7 Bläßle, A. *et al.* Quantitative diffusion measurements using the open-source software PyFRAP. *Nat Commun* **9**, 1582 (2018).

8 Levesque, S. G., Lim, R. M. & Shoichet, M. S. Macroporous interconnected dextran scaffolds of controlled porosity for tissue-engineering applications. *Biomaterials* **26**, 7436-7446 (2005).

9 Thorne, R. G., Lakkaraju, A., Rodriguez-Boulan, E. & Nicholson, C. In vivo diffusion of lactoferrin in brain extracellular space is regulated by interactions with heparan sulfate. *Proc Natl Acad Sci U S A* **105**, 8416-8421 (2008).

10 Netti, P. A., Berk, D. A., Swartz, M. A., Grodzinsky, A. J. & Jain, R. K. Role of extracellular matrix assembly in interstitial transport in solid tumors. *Cancer Res* **60**, 2497-2503 (2000).

11 Braeckmans, K., Peeters, L., Sanders, N. N., De Smedt, S. C. & Demeester, J. Three-dimensional fluorescence recovery after photobleaching with the confocal scanning laser microscope. *Biophys J* **85**, 2240-2252 (2003).

12 Segur, J. B. & Oberstar, H. E. Viscosity of glycerol and its aqueous solutions. *Industrial & Engineering Chemistry* **43**, 2117-2120 (1951).

13 Crank, J. *The Mathematics of Diffusion*. (Oxford Univ Press, Oxford, 1975).

14 Pinsky, P. M. Three-dimensional modeling of metabolic species transport in the cornea with a hydrogel intrastromal inlay. *Invest Ophthalmol Vis Sci* **55**, 3093-3106 (2014).

15 Fahrbach, F. O., Voigt, F. F., Schmid, B., Helmchen, F. & Huisken, J. Rapid 3D light-sheet microscopy with a tunable lens. *Opt Express* **21**, 21010-21026 (2013).

16 Power, R. M. & Huisken, J. A guide to light-sheet fluorescence microscopy for multiscale imaging. *Nat Methods* **14**, 360-373 (2017).

17 Jonsson, P., Jonsson, M. P., Tegenfeldt, J. O. & Hook, F. A method improving the accuracy of fluorescence recovery after photobleaching analysis. *Biophys J* **95** (2008).

18 Shi, C., Cisewski, S. E., Bell, P. D. & Yao, H. Measurement of three-dimensional anisotropic diffusion by multiphoton fluorescence recovery after photobleaching. *Ann Biomed Eng* **42**, 555-565 (2014).

19 Einstein, A. On the motion of small particles suspended in liquids at rest required by the molecular-kinetic theory of heat. *Annalen der physik* **17**, 549-560 (1905).
