# Supplementary material for: A noninvasive fluorescence imaging-based platform measures 3D anisotropic extracellular diffusion
Source: Nat Commun. 2021 Mar 26;12:1913. doi: 10.1038/s41467-021-22221-0 (PMC7997923; doi:10.1038/s41467-021-22221-0)
Supplement: Supplementary file 2 — Reporting Summary [file 41467_2021_22221_MOESM2_ESM.pdf]

nature research

Corresponding author(s): Tong Ye, Hai Yao

Last updated by author(s): 02/21/2021

Reporting Summary

Nature Research wishes to improve the reproducibility of the work that we publish. This form provides structure for consistency and transparency in reporting. For further information on Nature Research policies, see our Editorial Policies and the Editorial Policy Checklist.

Statistics

For all statistical analyses, confirm that the following items are present in the figure legend, table legend, main text, or Methods section.

n/a	Confirmed	
☐	☒	The exact sample size (*n*) for each experimental group/condition, given as a discrete number and unit of measurement
☐	☒	A statement on whether measurements were taken from distinct samples or whether the same sample was measured repeatedly
☐	☒	The statistical test(s) used AND whether they are one- or two-sided<br>*Only common tests should be described solely by name; describe more complex techniques in the Methods section.*
☐	☒	A description of all covariates tested
☐	☒	A description of any assumptions or corrections, such as tests of normality and adjustment for multiple comparisons
☐	☒	A full description of the statistical parameters including central tendency (e.g. means) or other basic estimates (e.g. regression coefficient) AND variation (e.g. standard deviation) or associated estimates of uncertainty (e.g. confidence intervals)
☐	☒	For null hypothesis testing, the test statistic (e.g. *F*, *t*, *r*) with confidence intervals, effect sizes, degrees of freedom and *P* value noted<br>*Give P values as exact values whenever suitable.*
☒	☐	For Bayesian analysis, information on the choice of priors and Markov chain Monte Carlo settings
☒	☐	For hierarchical and complex designs, identification of the appropriate level for tests and full reporting of outcomes
☒	☐	Estimates of effect sizes (e.g. Cohen's *d*, Pearson's *r*), indicating how they were calculated

Our web collection on statistics for biologists contains articles on many of the points above.

Software and code

Policy information about availability of computer code

Data collection	1. Labview 2014 (32 bit) - 3D FRAP data acquisition<br>2. Leica Application Suite (Version 2.6.3) - 2D FRAP data acquisition<br>3. Olympus Fluoview (Version 4.2a) - Second harmonic generation imaging<br>4. Olympus cellSens Standard 1.18 - Polarization and bright field imaging<br>5. Hitachi S-3700N (Version 7.16) - Scanning electron microscope imaging
Data analysis	1. Matlab 2017a - FRAP data processing and analysis<br>2. ImageJ (Version 1.52a) - Fiber alignment (coherency coefficient) analysis<br>3. Imaris (Version 9.3.1) - Generating 3D images from LiFT-FRAP data set<br>4. Adobe Photoshop CC 2015.5 - Generating all of the videos<br>5. Origin 2018 - Generating all of the graphs<br>6. COMSOL Multiphysics 5.4 - Modeling molecular dynamics and concentration distributions<br>7. Tecplot 360 2010 - Plotting simulation results<br>8. SPSS (Version 24.0) - Statistical analysis

For manuscripts utilizing custom algorithms or software that are central to the research but not yet described in published literature, software must be made available to editors and reviewers. We strongly encourage code deposition in a community repository (e.g. GitHub). See the Nature Research guidelines for submitting code & software for further information.

Data

Policy information about availability of data

All manuscripts must include a data availability statement. This statement should provide the following information, where applicable:

- Accession codes, unique identifiers, or web links for publicly available datasets
- A list of figures that have associated raw data
- A description of any restrictions on data availability

All resources and raw image data are available from the authors upon reasonable request. Source data for the figures are provided with this paper.

Field-specific reporting

Please select the one below that is the best fit for your research. If you are not sure, read the appropriate sections before making your selection.

- [x] Life sciences
- [ ] Behavioural & social sciences
- [ ] Ecological, evolutionary & environmental sciences

For a reference copy of the document with all sections, see nature.com/documents/nr-reporting-summary-flat.pdf

Life sciences study design

All studies must disclose on these points even when the disclosure is negative.

Sample size	Sample sizes were estimated based on our previously published FRAP studies (Shi et al. 2010; Shi et al. 2013; Shi et al. 2014). The sample size of each experiment was stated in the figure caption.
Data exclusions	No data was excluded.
Replication	All measurements were successfully repeated in at least 4 independent samples.
Randomization	Scaffolds, rat tail tendons, and porcine corneas were randomly allocated to different groups.
Blinding	Data collection and analysis were automatically performed through the softwares. Blinding was not applicable.

Reporting for specific materials, systems and methods

We require information from authors about some types of materials, experimental systems and methods used in many studies. Here, indicate whether each material, system or method listed is relevant to your study. If you are not sure if a list item applies to your research, read the appropriate section before selecting a response.

Materials & experimental systems

n/a	Involved in the study	
x		Antibodies
x		Eukaryotic cell lines
x		Palaeontology and archaeology
	x	Animals and other organisms
x		Human research participants
x		Clinical data
x		Dual use research of concern

Methods

n/a	Involved in the study	
x		ChIP-seq
x		Flow cytometry
x		MRI-based neuroimaging

Animals and other organisms

Policy information about studies involving animals; ARRIVE guidelines recommended for reporting animal research

Laboratory animals	3-month-old Sprague Dawley rats and 6-month-old Yorkshire pigs
Wild animals	This study did not involve wild animals
Field-collected samples	This study did not involve samples collected from the field
Ethics oversight	The use of animal tissues was approved by Clemson University and Medical University of South Carolina. Rat tails were collected at the conclusion of other IACUC approved research projects at the Medical University of South Carolina. Porcine eyeballs were obtained from a local abattoir. No animals were specifically sacrificed for this study.

Note that full information on the approval of the study protocol must also be provided in the manuscript.
