# Supplementary material for: A noninvasive fluorescence imaging-based platform measures 3D anisotropic extracellular diffusion
Source: Nat Commun. 2021 Mar 26;12:1913. doi: 10.1038/s41467-021-22221-0 (PMC7997923; doi:10.1038/s41467-021-22221-0)
Supplement: Supplementary file 9 — Description of Additional Supplementary Files [file 41467_2021_22221_MOESM9_ESM.pdf]

Title: Supplementary Movie 1:
Description: 3D LiFT-FRAP of sodium fluorescein in 55% glycerol solution. The volume was 76 µm × 76 µm × 76 µm and volume rate was 8 Hz. A total of 30 volumes of normalized postbleaching images were displayed.

Title: Supplementary Movie 2:
Description: 3D LiFT-FRAP of sodium fluorescein in 80% glycerol solution. The volume was 76 µm × 76 µm × 76 µm and volume rate was 8 Hz. A total of 76 volumes of normalized postbleaching images were displayed.

Title: Supplementary Movie 3:
Description: 3D LiFT-FRAP of FD10 in 60% glycerol solution. The volume was 76 µm × 76 µm × 76 µm and volume rate was 8 Hz. A total of 76 volumes of normalized postbleaching images were displayed.

Title: Supplementary Movie 4:
Description: 3D LiFT-FRAP of FD20 in in-situ, native porcine cornea. The volume was 76 µm × 76 µm × 76 µm and volume rate was 8 Hz. A total of 40 volumes of normalized postbleaching images were displayed.

Title: Supplementary Movie 5:
Description: 3D LiFT-FRAP of sodium fluorescein in healthy rat tail tendon. The volume was 76 µm × 76 µm × 76 µm and volume rate was 8 Hz. A total of 70 volumes of normalized postbleaching images were displayed.

Title: Supplementary Movie 6:
Description: 3D LiFT-FRAP of FD20 in fiber-aligned scaffold. The volume was 76 µm × 76 µm × 76 µm and volume rate was 8 Hz. A total of 76 volumes of normalized postbleaching images were displayed.
